# Genetic and molecular breeding perspectives on developing abiotic stress tolerant pea (*Pisum sativum* L.)

**DOI:** 10.3389/fpls.2026.1762081

**Published:** 2026-03-10

**Authors:** Neha Verma, Rajinder Kumar Dhall, Saurabh Yadav, Neha Rana, Manpreet Kaur, Priyanka Kumari, Parteek Kumar, Rishabh Maheshwari, Priti Sharma

**Affiliations:** 1 GS Khush Institute of Genetics, Plant Breeding and Biotechnology, Punjab Agricultural University, Ludhiana, India; 2 Department of Vegetable Science, Punjab Agricultural University, Ludhiana, India; 3 School of Agricultural Biotechnology, Punjab Agricultural University, Ludhiana, India; 4 Department of Plant Pathology, Punjab Agricultural University, Ludhiana, India

**Keywords:** abiotic stress, genetic, molecular breeding, next generation strategies, OMICS techniques, pea

## Abstract

Pea is an important cool-season legume crop of the genus *Pisum* used for food and feed due to its high protein content. Pea plants often experience limitations in their potential productivity due to various abiotic stresses such as heat, drought, salinity and frost. These stresses exhibit complex quantitative inheritance, rendering conventional breeding slow and laborious due to long generation cycles. This underscores the need for genomic accelerated breeding approaches. Therefore, this review provides detailed insights into significant abiotic stresses affecting pea yield, available genetic resources, tolerant genotypes developed, and genomic advancements like reference genome information, QTL mapping, GWAS, transcriptomics, proteomics, and transgenics. These techniques enabled the identification of stress-tolerant gene(s) within the pea gene pool and their introgression into elite varieties to accelerate genetic gains in a breeding program. Additionally, advances and accomplishments achieved using cutting-edge techniques viz., gene editing, speed breeding, phenomics, genomic selection, epigenetic breeding, functional marker development and allele mining are discussed as time-efficient strategies for developing novel pea phenotypes resilient to abiotic stress. Integrating conventional breeding with advanced molecular and modern omics techniques will revolutionize the pea abiotic stress tolerance breeding program.

## 1 Introduction

Pea (*Pisum sativum* L.) is a vital leguminous crop highly valued for its nutritional content and role in crop rotation (Dhall et al., 2023). Peas are a primary dietary protein source in Asia and Africa (Verma et al., 2025). Beyond its agronomic importance, pea plays a significant role in global food and nutritional security. It serves as an affordable source of high-quality plant protein (20–25%), dietary fiber, essential amino acids, vitamins, and minerals. The crop also contributes to sustainable agriculture through biological nitrogen fixation, improving soil fertility, and reducing dependence on synthetic nitrogen fertilizers. In India, pea cultivation supports smallholder farmers and the processing industry, thereby contributing to rural livelihoods and value chains. The growing global demand for plant-based protein further underscores the strategic importance of enhancing pea productivity under climate-resilient production systems. Globally, pea productivity increased only marginally from about 78.7 q/ha in 1994 to 80.9 q/ha in 2023 (FAOSTAT, 2024), highlighting the stagnation in yield improvement despite advances in cultivar development. This limited productivity gain is largely attributed to the rising impact of abiotic stress exacerbated by global climate change, which affects flowering, pod set, and seed filling. The increasing prevalence of heat, drought, frost, salinity, and early-season waterlogging has therefore made stress tolerance breeding a key priority for improving pea productivity in India (Warkentin et al., 2015). These stresses vary by region and growing type, making it difficult for breeders to develop broad, adaptable, high-yielding cultivars (Tayeh et al., 2015b) Thus, breeding peas for tolerance against abiotic stresses through advanced breeding strategies is crucial for ensuring sustainable food production and nutritional security in the current global challenges of increasing population pressure and shifting climatic patterns (Tayeh et al., 2015a; Tayeh et al., 2015b; Tayeh et al., 2015c). Historically, breeding efforts in legumes, including peas, have lagged behind cereals, leaving a significant opportunity to enhance pea productivity (Sultana et al., 2014).

**Figure 1:**
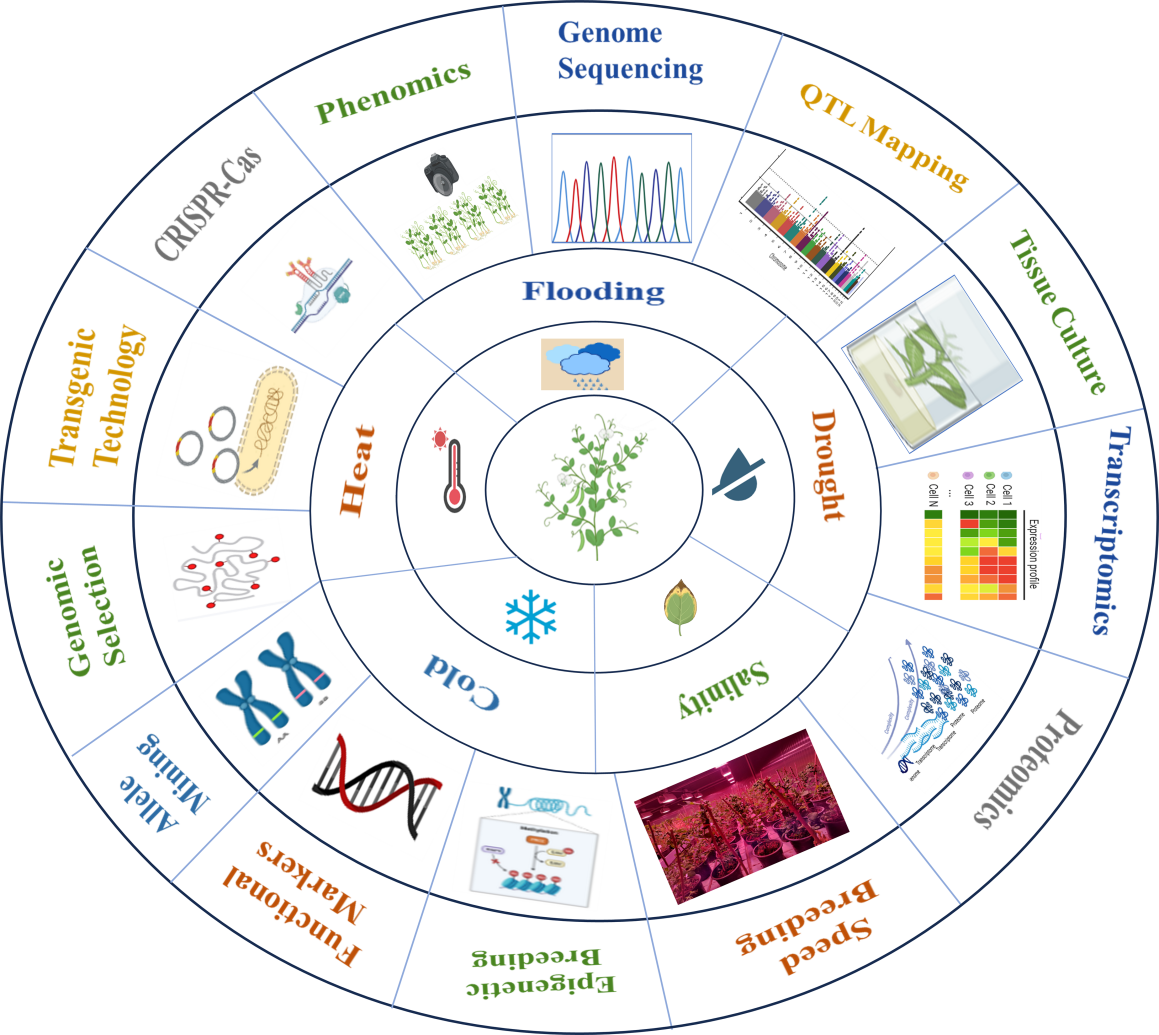
Integrated molecular breeding strategies to enhance abiotic stress tolerance in pea.

Improving pea tolerance to abiotic stresses remains a primary objective for breeders and geneticists. Although *Pisum* species harbor significant genetic diversity based on biological status, geographical regions, and morpho-agronomic traits (Abdurakhmonov and Abdukarimov, 2008). Genetic gain through traditional breeding approaches is slow due to multigenic inheritance, strong genotype×environment (G×E) influence and long generation cycles. These limitations require a need for an integrated breeding, genomic and biotechnological approach (Ahmad et al., 2021; Varshney et al., 2019). Therefore, a holistic approach that combines diverse germplasm, advanced breeding technologies, and stress-resilient management practices is necessary to achieve genetic gains. Various techniques like marker-assisted selection (MAS) accelerate the introgression of specific stress-related genes from wild relatives or landraces into elite varieties (Tayeh et al., 2015b). Genomic selection uses genome-wide markers to predict performance for complex traits and speed up the breeding cycles (Crossa et al., 2017), while omics technologies help to identify key metabolic pathways involved in stress response (Smýkal et al., 2017). Likewise, genetic engineering introduces stress-responsive genes such as those regulating proline accumulation. The use of advanced CRISPR/Cas9 genome-editing techniques also enables precise modifications of stress-responsive genomic regions (Borrelli et al., 2018). Together, these integrated approaches enable efficient and precise breeding of stress-tolerant pea varieties. While several previous reviews have discussed individual abiotic stresses or specific molecular tools in pea, a comprehensive synthesis linking stress physiology, genetic resources, and modern breeding technologies within a climate-resilient framework remains limited. This review therefore provides an integrated and up-to-date perspective on the major abiotic stresses affecting pea, evaluates available genetic variability, and critically examines advances in marker-assisted selection, genomic selection, omics approaches, and genome editing. By bridging conventional and next-generation breeding strategies, this review aims to offer a structured roadmap for accelerating the development of stress-resilient pea cultivars suited to diverse agro-ecological environments (**Figure 1**).

## 2 Types of abiotic stress in pea

Peas are sensitive to multiple abiotic stresses, including heat, drought, frost, salinity, and waterlogging. Among these, drought, waterlogging, and heat cause the most significant yield losses, while salinity, alkalinity, and frost pose relatively minor concern (Sultana et al., 2014). Heat stress during critical growth phases like germination and flowering leads to 20-70% yield losses (Devi et al., 2023). At 38°C temperature plant showed a decline in total phenolics, free proline, and hydrogen peroxide content, followed by an increase in superoxide dismutase, guaiacol peroxidase, and catalase activity (Todorova et al., 2016). Molecularly, heat shock proteins (HSPs) such as *HSP70* and *HSP90* protect cells by stabilizing and refolding damaged proteins. Heat shock transcription factors (HSFs) regulate the HSP expression and enhance heat tolerance (Devi et al., 2023). These HSPs also contribute to membrane stability and osmolyte accumulation helping maintain cellular hydration under dehydration. However, under combined heat and drought stress these molecular defenses fail to sustain membrane integrity and osmolyte homeostasis, directly causing tissue dehydration, leaf senescence, disrupting cellular redox balance, reduced turgor pressure and impaired cellular and tissue development that limits productivity (Bagri and Janeja, 2021). Beyond combined stress effects, drought alone causes significant yield reductions ranging from 21–54%, particularly when stress coincides with flowering and pod-filling stages (Baigorri et al., 1999; Červenski et al., 2017). Drought alters carbohydrate metabolism and protein–starch ratios, thereby affecting seed development. In legumes, drought stress further limits productivity by impairing biological nitrogen fixation due to reduced leghemoglobin content, nodule number, and nitrogen assimilation efficiency (Rana et al., 2016).

Pea also shows greater sensitivity to waterlogging than cereals and oilseeds (Rana et al., 2016). In pea, waterlogging increases soil-borne fungal diseases risk, promotes anaerobic bacterial activity, which converts sulfate (SO_4_²^-^) to hydrogen sulfide (H_2_S) (Rana et al., 2016) and inhibits root growth and metabolism (Pastor et al., 2017). Additionally, waterlogging adaptive cultivars maintain stronger root metabolic activity and induce anaerobic stress responsive enzymes such as alcohol dehydrogenase (*ADH*) and cellulase, while testa integrity serves as an indirect selection method for waterlogging tolerance in *Pisum sativum* var. *arvense* (Kaur and Bedi, 2011, Zaman et al., 2019). Pea cultivation is adversely affected under high salinity conditions, as excessive salt accumulation disrupts plant metabolism by reducing water potential, causing ion toxicity and impairing CO_2_ assimilation (Ehtaiwwesh and Emsahel, 2020). The initial stages of growth, particularly seed germination and early seedling development are especially vulnerable to salt stress. Salinity hampers these stages by inducing osmotic stress, disrupting ion homeostasis, and generating oxidative damage (Ehtaiwwesh and Emsahel, 2020). Several physiological and biochemical adaptations are involved to combat the toxic effects of sodium ions (Na+) and oxidative stress. The salt-tolerant pea activates a robust antioxidant defense system with increased key enzymes viz., ascorbate peroxidase (*APX*), glutathione reductase (*GR*), and dehydroascorbate reductase (*DHAR*) activity (Hernandez et al., 2000). Additionally, the upregulation of genes such as mitochondrial *Mn-SOD*, *cytosolic APX*, and *chloroplastic CuZn-SOD* further supports the plant’s ability to endure prolonged salt stress (Hernandez et al., 2000). Frost is a significant threat in temperate regions where freezing temperatures inhibit photosynthesis due to moisture stress, internal injury and the production of ROS (Bhat et al., 2022). Frost and chilling stress during early growth stages can result in seedling death, reduced adaptability, and reproductive challenges, such as bud, flower, and pod abortion (Liu et al., 2017). Thus, developing breeding winter-hardy varieties and identifying frost-tolerance genes are critical objectives for future research (Zong et al., 2019). During cold stress, plants enhance antioxidant enzyme activity like catalase (CAT), superoxide dismutase (SOD), and APX to reduce oxidative damage caused by ROS (Shahid et al., 2012). Molecular responses include activation of cold-responsive genes and transcription factors like *CBF/DREB*, which regulate the production of stress-induced proteins on exposure to cold (Bhat et al., 2022). Additionally, accumulating osmoprotectants such as proline and sugars helps maintain cellular function and prevent water loss under cold conditions (Kovács et al., 2011). Thus, collectively different stresses of pea exhibit conserved physiological and biochemical response mechanisms. These include ROS accumulation, osmolyte accumulation (proline, soluble sugars), electrolyte leakage, antioxidant enzyme activation (SOD, catalase and peroxidase) and induction of HSP expression for protein stabilization. The convergence of these responses across stresses highlights the potential for developing multi-stress tolerant pea genotypes through integrated breeding approaches.

## 3 Genetic improvement of pea toward abiotic stress tolerance

Genetic improvement is essential for enhancing pea crop resilience to abiotic stress, such as heat, drought, salinity, and frost. Traditional breeding approaches alone are insufficient to overcome these challenges. The genetic diversity within pea germplasm, coupled with modern genomic tools, provides an opportunity to develop cultivars that can thrive under challenging environmental conditions (Tayeh et al., 2015b; Coyne et al., 2020). This is important, especially in regions where climate extremes are becoming more frequent, and crop adaptability is becoming crucial for long-term sustainability. Breeders aim to enhance the ability of pea to withstand abiotic stresses by improving the genetic makeup of pea plant, thereby securing its role as a vital leguminous crop worldwide (Smýkal et al., 2017). Efforts have been made to develop abiotic stress-tolerant pea genotypes based on plant stress response phenotyping (**Table 1**). However, genetic improvement is limited by the complex and multigenic nature of stress tolerance trait(s) (Kumar et al., 2011). Different stress-tolerant cultivars have been bred despite complexity via trait introduction from stress-tolerant wild relatives or landraces (Bartels and Sunkar, 2005). Most landraces hold significant potential to impart abiotic stress tolerance, while wild pea species contribute only 2% of the worldwide collection (Smýkal et al., 2017). Wild species serve as valuable donors for various traits due to the absence of crossing barriers with the cultivated pea.

**Table 1:** List of genotypes tolerant to different abiotic stresses.

Stress	Variety/Genotypes tolerant	Origin	References
Heat	Mater Ageta-6	India	Mohan et al., 2013
	Early Badger	USA	Mohan et al., 2013
	Mater Ageta-7	India	Dhall and Brar, 2015
	CDC Centennial	Canada	Huang et al., 2017
	Arka Chaitra, Arka Tapas, Arka Uttam	India	Susmita et al., 2020
	IPFD 11-5,	–	Lamichaney et al., 2021
	Pant P-72, JP-625, IARI-2877	India	Lamichaney et al., 2021
	P-1544-1, HUDP 11, PMR-38 I	–	Lamichaney et al., 2021
	IIHR 544	India	Sharma et al., 2023
Drought	Early Badger	USA	Mohan et al., 2013; Sharma et al., 2023
	P665	–	Iglesias-García et al., 2015
	Vivek Matar-6	India	Sharma et al., 2023
Frost	Freezer, Alderman, Taichung – 12	–	Mohan et al., 2013; Sharma et al., 2023
	KI_L38, KA_37, Isard	–	Liu et al., 2017
	Dolmen, Champagne	France	Liu et al., 2017
	SHAN WAN DOU 8, 11, 12, 13, DA BO GE HUI, QI DONG CAO WAN DOU, MA WAN DOU	China	Liu et al., 2017
	K-129	Greece	Liu et al., 2017
	921329-1	–	Liu et al., 2017
	Arkel	–	Sharma et al., 2023
	ATC 104	United Kingdom	Sharma et al., 2023
	ATC 377	Estonia	Sharma et al., 2023
	ATC 968	Italy	Sharma et al., 2023
	ATC 3992	Kazakhstan	Sharma et al., 2023
	ATC 4204	China	Sharma et al., 2023
	Vivek Matar-6	India	Sharma et al., 2023
Salinity	Climax, Samarinazard, 9800-5	Pakistan	Shahid et al., 2012
	New line perfection, Market prize	–	Sharma et al., 2023
	ATC1836	Greece	Sharma et al., 2023
Waterlogging	Semi-Leafless	–	Gonzalez et al., 2002

A deep-rooted landrace from Ethiopia (JI1432) has been well adapted to a wide temperature range, primarily due to its robust root system, which improves water uptake efficiency, and its phenological plasticity that allows stable growth across variable thermal regimes (Araújo et al., 2015). Additionally, *Pisum fulvum* is a potential source of drought tolerance, as its primary root penetrates deeper into the soil rapidly, enabling access to residual soil moisture under water-deficit conditions (Naim-Feil et al., 2017). Furthermore, favorable root system architecture (RSA) traits in *P. fulvum* and waxy leaf surfaces in the *Pisum* genus contribute to reduced transpirational water loss and enhanced drought adaptation (Naim-Feil et al., 2017). In *P. abyssinicum*, early flowering has been exploited as a drought escape mechanism by allowing completion of the life cycle before the onset of severe moisture stress (Coyne et al., 2020). Similarly, Huang et al. (2017) identified a wild pea genotype, HR1, adapted to drought-prone environments in which tolerance was associated with superior biomass maintenance, delayed stress-induced senescence and improved yield stability under limited water availability. In India, specific local pea landraces such as Magadi Local, Shihara Local (VRPSel-1), and Kasmiria are cultivated by farming communities under heat-stress conditions, reflecting their inherent adaptation through heat tolerance, phenological adjustment and farmer-led selection (Devi et al., 2023). *P. elatius* accessions are also adapted to low-temperature environments (Ali et al., 1994). Furthermore, pea crop wild relatives represent a valuable gene pool for developing adaptation to a wide range of abiotic stresses (Coyne et al., 2020). Additionally, more than 73,931 accessions conserved across over 28 national and international collections provide a vast genetic reservoir for breeding stress-tolerant pea varieties (Smolikova et al., 2020). Despite the availability of extensive germplasm resources, their effective utilization for specific abiotic stress tolerance remains limited. Major gaps include insufficient high-throughput and multi-environment phenotyping, limited genomic characterization of diverse accessions, inadequate pre-breeding efforts to introgress useful alleles into elite genetic backgrounds, linkage drag and weak integration between germplasm repositories and active breeding programs. Furthermore, combined stresses (e.g., heat–drought or salinity–waterlogging) also lack systematic screening of global collections. Modern genomic approaches like next generation sequencing, high-throughput genotyping, genome sequencing and genomic selection can help address these gaps by dissecting complex stress-responsive traits and accelerating allele deployment. Strengthened collaboration between gene banks and breeding programs coupled with phenomics and genomics-assisted pre-breeding will translate conserved diversity into climate-resilient cultivars. Thus, the transition from conventional germplasm characterization to genomics-assisted breeding represents a critical step toward climate-resilient pea cultivar development.

## 4 Genomic resources and approaches for dissecting abiotic stress tolerance

High-quality reference genome and gene annotation facilitate the characterization of genetic traits and serve as fundamental genomic resources (Coyne et al., 2020). The first chromosome-level sequence draft assembly of *Pisum sativum* L. inbred accession “Cameor” was generated. The estimated pea genome size is around 4.45 Gb, out of which 3.92Gb (88%) of the genome is assemble (Kreplak et al., 2019). Different research groups sequenced the genomes of different accessions in pea (**Table 2**). The draft assembly of inbred pea accession Zhongwan6 was constructed in 2022, revealing an estimated genome size of 3.8 Gb. In Feb 2024, the genome of inbred pea accession Zhewan No. 1 was also sequenced with an estimated genome size of 3.9 Gb. The advancement of genomic resources facilitates a precise and accurate understanding of genetic architecture, trait mapping, gene identification and molecular marker development in pea. The availability of chromosome level assemblies across diverse accessions enabled accurate interpretation of stress responsive traits supporting the application of genomic assisted breeding for improved productivity and climate resilience.

**Table 2:** Summary of reference genome assemblies for *Pisum sativum*.

Assembly name	Variety	GenBank assembly name	Year	Level	Scaffold count	Genome size	Genome coverage	Gene	Protein	Bio project number	Source
ASM301357v1	Gradus No 2	GCA_003013575.1	2018	Scaffold	5,449,423	4.3 Gb	86.0x	–	–	PRJNA432052	https://www.ncbi.nlm.nih.gov/datasets/genome/GCA_003013575.1/
*Pisum_sativum*_v1a	–	GCA_900700895.2	2019	Chromosome	14,273	3.9 Gb	150.0x	–	–	PRJEB31320	https://www.ncbi.nlm.nih.gov/datasets/genome/GCA_900700895.2/
PSA_r1.0	JI128	GCA_014905515.1	2020	Contig	–	3.3 Gb	44.0x	–	–	PRJDB10540	https://www.ncbi.nlm.nih.gov/datasets/genome/GCA_014905515.1/
CAAS_Psat_ZW6_1.0	Zhongwan6	GCF_024323335.1	2022	Chromosome	1566	3.8 Gb	85.2x	65,672	40,025	PRJNA730094	https://www.ncbi.nlm.nih.gov/datasets/genome/GCF_024323335.1/
ASM2453087v1	Frisson	GCA_024530875.1	2022	Scaffold	2254	3.7 Gb	100.0x	–	–	PRJNA853105	https://www.ncbi.nlm.nih.gov/datasets/genome/GCA_024530875.1/
*Pisum sativum* CEN6	–	GCA_947076115.1	2022	Contig	–	177.6Mb	45.0x	–	–	PRJEB54858	https://www.ncbi.nlm.nih.gov/datasets/genome/GCA_947076115.1/
ASM3678607v1	Zhewan No.1	GCA_036786075.1	2024	Chromosome	2906	3.9 Gb	40.0x	–	–	PRJNA1042956	https://www.ncbi.nlm.nih.gov/datasets/genome/GCA_036786075.1/
JIC_Psat_v1.3	JI2822	GCA_964186695.1	2024	Chromosome	170	3.8 Gb	23.0x	33,559	33,554	PRJEB75659	https://www.ncbi.nlm.nih.gov/datasets/genome/GCA_964186695.1/
JIC_PsatJI15_v1.3	JI15	GCA_964214105.1	2024	Chromosome	86	3.9 Gb	25.0x	–	–	PRJEB78909	https://www.ncbi.nlm.nih.gov/datasets/genome/GCA_964214105.1/

### 4.1 Unravelling the genetic complexity of abiotic stress response through genomics

Breeding pea varieties with enhanced tolerance to abiotic stresses has become a crucial objective for plant breeders. Recent advances have improved our understanding of the genetic mechanisms underlying abiotic stress tolerance through the identification of QTLs, candidate genes and key proteins involved in stress response (**Tables 3**, **4**). These genomic discoveries enable the transfer of tolerance-associated regions into elite breeding backgrounds using molecular marker (Ahmad et al., 2020). Additionally, the Pea Marker Database (PMD) compiles gene-based markers along with their linkage group positions and corresponding transcript sequence (Tayeh et al., 2015b; Kulaeva et al., 2017). This database consists of PMD1 and PMD2 including 2,484 and 15,944 genetic markers, respectively, their positions in linkage groups and corresponding pea transcript sequences.

**Table 3:** QTL mapping for abiotic stress tolerance in *Pisum sativum*.

Abiotic stress	Cross	Population	QTLs identified	References
Heat	CDC Centennial × CDC Sage	RIL	10 QTLs under different environment for various traits	Huang et al., 2017
	PR11-2×CDC Amarillo	RIL	Genomic mapping for heat-responsive traits identified four stable QTLs	Huang et al., 2023
Frost	Champagne×Terese	RIL	Stable QTL: LG V and LGVI under different environments	Dumont et al., 2009
	Champagne×Terese	RIL	Six QTL and most significant QTL LGIII	Weller et al., 2012
	Champagne×Terese	RIL	3 QTL	Legrand et al., 2013
	China(JI1491)× Caméor.	RIL	LGIII, LG V, LG VI,	Klein et al., 2014
	Three biparental population	RIL, Meta QTL	Five Meta QTL on LGIII, LG V, LG VI	Boutet et al., 2023
Salinity	Kaspa (Sensitive)×Parafield (Moderately tolerant)	RIL	LGIII, LGVII	Javid et al., 2015

**Table 4:** Genome wide association studies for abiotic stress tolerance in *Pisum sativum*.

Stress	Pea accession/panel	Technique	Marker trait association (MTA)/Linkage group	Gene(s) identified	References
Heat	135 pea accessions	GWAS	15 MTAs	16 candidate genes	Tafesse et al., 2021
	94 pea accessions	GWAS	26 MTA and two SNPs on Chr 2 & 6 co-localize with genes	19 candidate genes	Zanetta et al., 2024
	137 pea accessions	GWAS	32 MTA	48 candidate genes	Tafesse et al., 2020
Drought	135 pea accessions	GWAS	15 MTAs,	16 candidate genes;	Tafesse et al., 2021
	315 pea lines	GBS of three RIL populations	26 marker	–	Bagheri et al., 2023
Frost	365 pea accessions	GWAS	LG I, LG II, LG III, LG V, LG VI, LG VII	C-repeat Binding Factors (*CBF*)	Beji et al., 2020
	672 pea diverse panel	Association analysis with 267 SSR markers	Significant seven markers, Marker EST1109 on LG VI co-localized with gene	Gene involved in glycoprotein metabolism	Liu et al., 2017
	917 pea accessions	Association analysis with ~13,000 SNPs	LG5 and LG6	–	Siol et al., 2017
Salinity	311 accessions	GWAS with 68,222 SNPs	LG II, LGIV, LGVI, LGVII;	SNPs showing strong association: S6LG2_20798185, S4LG4_126644638	Tracy, 2020

#### 4.1.1 QTL mapping

A RIL (Recombinant Inbred Line) population derived from cross CDC Centennial × CDC Sage was evaluated under normal and late seeding conditions, with the latter simulating heat stress on flowering and yield traits. A genomic linkage map with 1024 loci covering 1702 cM genetic distance identified 10 QTLs across environments (Huang et al., 2017). Another RIL panel (PR-24) evaluated under normal and heat-stress field conditions reported plant height as a consistent yield predictor across environments. Four stable QTLs were also mapped for different traits, and two indices, viz., geometric mean yield (GMP) and stress tolerance index (STI) were effective as selection tools for breeding heat-resilient, high-yielding pea cultivars (Huang et al., 2023). Furthermore, assessment of drought symptoms along with relative water content in soil (RWCS) and leaves (RWCL) within the RIL population identified ten QTLs associated with drought adaptation which explained 9-33% trait wise phenotypic variation (Iglesias-García et al., 2015). Frost tolerance has become another key focus for pea breeders. RIL population derived from Champagne × Terese cross was assessed using a frost damage scale and QTL linked to frost tolerance was identified on several chromosomal regions, which explained 6.5 to 46.5% phenotypic variation. Notably, QTL on linkage groups 5 and 6 were consistent across all experiments (Dumont et al., 2009). Flowering locus *Hr* is believed to impact winter frost tolerance in pea by delaying flowering until after critical freezing periods (Klein et al., 2014). The genetic basis for frost tolerance in 164 RIL population of Champagne × Terese cross was evaluated and six QTLs were identified. A significant QTL of pea frost tolerance on LGIII was located near the *Hr* locus (Weller et al., 2012). Recently, 62 SNP markers were identified linked to frost tolerance in winter pea through a GWAS of 365 accessions. It also confirmed three previously mapped QTLs and discovered new loci on LG II, I and VII, revealing candidate genes including C-repeat Binding Factors (*CBF*) that could enhance breeding for frost tolerance (Beji et al., 2020). Five metaQTLs (*MDAF.3.1, MDAF.3.2, MDAF.5.1, MDAF.5.2, and MDAF.6.2*) were also identified across three linkage group (LG III, LG V and LG VI) in pea that regulate frost tolerance (Boutet et al., 2023). For salinity tolerance, RIL population derived from cross Kaspa (Sensitive) × Parafield (Moderately tolerant) identified QTLs on linkage groups III (*PsIII-QTL1*) and VII (*PsVII-QTL2*). The candidate genes as *14-3-3-like protein*, *receptor-like protein kinase*, *glutamine synthetase*, and *histone deacetylase* associated with salt tolerance were identified. Additionally, the gene in the *PsIII-QTL1* region was annotated as a salt-tolerant protein (Javid et al., 2015).

#### 4.1.2 Genome-wide association studies

Determining genetic diversity and identifying valuable alleles is important for enhancing crop resilience and adaptability for breeding programs. GWAS is one of the widely used technique that help in identifying genetic markers linked to quantitative traits by leveraging linkage disequilibrium (LD) between candidate genes and genetic markers (Abdel-Hamid and Salem, 2021). Limited information is available on GWAS for abiotic stress tolerance in pea (**Table 4**). A GWAS on heat and drought adaptive traits constituting 135 pea accessions association panels was conducted using genotyping by sequencing (GBS) approach to assess different phenotypic traits across five environments (Tafesse et al., 2021). A 16,877 total SNPs were employed to establish marker-trait associations (MTAs), revealing that 15 markers were associated with different traits. Sixteen candidate genes were discovered within a 15 Kb distance on markers either side for stress-hardy pea cultivars (Tafesse et al., 2021). Genetic variation in Southeast Asia pea accessions was also studied and MTA was identified for heat stress. GWAS of 94 pea accessions from Southeast Asia for 11 different traits under high temperatures in Malaysia and Indonesia identified 1974 high-quality SNPs. Out of these SNPs, 26 SNPs showed significant MTA and two SNPs were identified on chromosomes 2 and 6 which co-localize with genes related to environmental stress responses. Additionally, 19 candidate genes were associated with different traits. The identified SNP markers and candidate genes have the potential for marker-assisted selection to develop heat-tolerant pea cultivars (Zanetta et al., 2024). Genotyping of 365 pea accessions of GWAS panel with Illumina Infinium^®^BeadChip to gather marker data for 11,366 SNPs. Out of these 11,366 SNPs, 62 SNPs were found to be linked with frost tolerance and distributed in six linkage groups. Sixteen QTLs on LGI, V, VI and VII for winter frost damage in multiple winter environments were identified and fifty candidate genes were associated with frost damage-related loci (Klein et al., 2014; Beji et al., 2020). Association mapping of 672 pea diverse panel using 267 SSR markers revealed seven markers significantly associated with frost tolerance. Notably, EST marker EST1109 on LG VI co-localized with a glycoprotein metabolism gene under chilling stress which offers novel insights for marker-assisted breeding (Liu et al., 2017). An association panel of 917 pea accessions was also genotyped using ∼13,000 SNPs and identified two differentiated regions on LG5 and LG6 comprising 33 SNPs which showed stronger divergence between winter and spring types. These regions overlap with previously reported QTLs for cold acclimation and frost tolerance (Siol et al., 2017). For frost tolerance, *CBF* transcription factors is a potential genetic determinant identified on LG VI with a *CBF* annotated marker reported in high linkage disequilibrium with frost damage associated loci (Beji et al., 2020). Furthermore, GWAS involving 68,222 SNPs identified 19 significant markers (P < 1 × 10^-6^) associated with salinity tolerance traits at germination and seedling stages in pea which distributed across chromosomes 1, 4, 6, and 7 (LGs 6, 4, 2, and 7). Notably, SNP ‘S6LG2_20798185’ was linked to both final score and AUIC traits, while ‘S4LG4_126644638’ showed the strongest association (P = 6.92 × 10^-^¹²) with percent plant height under salinity (Tracy, 2020). However still there is a research gap exists in pea compared to cereal crops where a large number of QTLs/genes has been identified. Cereal crops have particularly benefited from extensive meta QTL analysis (>500 abiotic stress QTLs mapped with high density SNP arrays), pea research lags with limited QTL identification. Thus, both QTL mapping and GWAS have substantially advanced abiotic stress tolerance genomics in pea. QTL mapping identifies major effect loci through biparental populations; however, its mapping resolution is limited by low recombination, narrow genetic diversity and high G×E instability (Li et al., 2010). Contrarily, GWAS enables broad allele mining across diverse germplasm but is sensitive to complex linkage disequilibrium patterns and population structure in small pea association panels (Clauw et al., 2025). The complementary integration of GWAS for fine scale allelic diversity and QTL mapping for validation offers a powerful strategy for dissecting complex polygenic traits such as heat, drought and frost tolerance.

Numerous QTLs and candidate genes have been identified for abiotic stress tolerance in pea (**Table 3** and **Table 4**). However, pea significantly trails major cereal crops (Wheat and maize), where >500 meta-QTLs, high-density SNP arrays and large multi-environment populations have enabled effective marker-assisted breeding and genomic selection for abiotic stress tolerance (Acuña-Galindo et al., 2015; Tanin et al., 2022; Sheoran et al., 2022). In contrast, pea research is constrained by smaller populations, lower marker density and limited GWAS panels (Tafesse et al., 2021; Zanetta et al., 2024), highlighting the need to adopt successful cereal strategies including large multi-parent populations, pan-genome resources and high-throughput phenotyping integrated with genomic selection.

Despite these advances, breeding translation of identified QTLs in pea is constrained by G×E interactions, multi-QTL pyramiding challenges and trade-off between stress tolerance and yield potential. Therefore, multi-environment validation across different climatic zones, together with the integration of marker-assisted and genomic selection leveraging pea’s genetic diversity to optimize stress tolerance without compromising yield for sustainable and durable genetic gains in pea.

### 4.2 Transcriptomics and proteomics

Pea genome availability significantly enhances the transcriptomic studies by providing a reference framework that facilitates candidate gene identification linked to desirable agronomic traits (**Table 5**). This allows researchers to unravel regulatory networks and molecular pathways critical for improving crop resilience and productivity (Afzal et al., 2020). Several candidate genes associated with heat tolerance have been identified in pea based on gene ontology (GO) (Huang et al., 2023; Kanwar et al., 2023; Sharma et al., 2022). The heat-induced genes were linked to biological processes such as heat response, protein folding and DNA transcription. The distinct responses of each variety highlight the complexity of heat tolerance mechanisms with specific processes like DNA damage response and electron transport chain activity observed in PR11–2 and CDC Amarillo (Huang et al., 2021). In pea, 38 *PsHsf* genes were identified and classified into three different classes A, B and C which were distributed across all seven chromosomes. Notably, *PsHsfA2a* (with isoforms *PsHsfA2aI, PsHsfA2aIII and PsHsfA2aII), PsHsfA3, PsHsfB1a*, *PsHsfA6b, PsHsfB2a* and *PsHsfA9* were significantly upregulated under heat, drought and salt stress, highlighting their potential roles as key regulators in abiotic stress responses (Kanwar et al., 2023).

**Table 5:** Genetic determinants of abiotic stress tolerance in *Pisum sativum*.

Abiotic stress	Genotype	Plant part used	NGS platform/Expression analysis	Gene(s)	References
Heat	PR11-2, PR11-90, CDC Amarillo	Heat stressed anthers and stipules	Illumina NovaSeq platform	Anther: 808 DEGs (588 up regulated and 220 downregulated); Stipule: 879 DEGs (463/416 upregulation/downregulation)	Huang et al., 2023
	–	Leave, flower and pod	Expression analysis	*PsHsf: PsHsfA2a isoforms, PsHsfA3, PsHsfA6b, PsHsfA9, PsHsfB1a, PsHsfB2a*	Kanwar et al., 2023
	P-89, Arkel, and Azad Pea-1	Seedling	Expression analysis	*ERF60, ERF92, HSFA2*	Sharma et al., 2022
Drought	Stirone	Root	Sequencing and Expression analysis	ABA-related pathway and ROS detoxification	Schillaci et al., 2024
	Prima	Embryonic axes	Illumina	ABA stimulus (*LTI65, LTP4, and HVA22E*)	Smolikova et al., 2020
	Frisson	Leaf	Illumina HiSeq4000	*P5CS2, ALDH12A1, SIP2, YUCCA6* & different transcription factors	Álvarez-Aragón et al., 2023
	Caméor	Seed	Illumina HiSeq3000	SUMO ligases	Henriet et al., 2019
	–	–	Expression analysis	*WRKY TFs* (*PsWRKY23, PsWRKY58, PsWRKY64*),	Jarambasa et al., 2023; Kumar et al., 2024
	TM-1	Leaf	Expression analysis	kinesin genes (*PsKIN8, PsKIN11, PsKIN54*), *MADS-box genes*	Yuan et al., 2024; Gong et al., 2025
Frost	Champagne & Terese	Soot, root and leaf	Microarray and Expression analysis	*CBF, COR and LEA*	Lucau-Danila et al., 2012
	Champagne & Terese	Seedling	Expression analysis	Glycine degradation pathway, jasmonate pathway, LEA proteins	Legrand et al., 2013
	Champagne & Terese	–	Illumina and Expression analysis	4489 DEGs	Bahrman et al., 2019
	Champagne & Terese	Seedling	Illumina HiSeq 2000/2500	11 miRNA families	Mazurier et al., 2022
	Champagne & Terese	Leaf	–	*PsMIF1-4, PsZHD1,PsZHD6, PsZHD10*	Shi et al., 2023
Salinity	Meteor, Green grass, Climax	Leaf	Expression analysis	*P5CR, POX, SOD, PAL1*	Farooq et al., 2024
	–	–	Expression analysis	*WRKY TFs* (*PsWRKY23, PsWRKY58, PsWRKY64*),	Kumar et al., 2024

Gene expression studies have also provided the valuable insights into drought stress response in pea (Schillaci et al., 2024). Transcriptomic changes during seed to seedling transition identified key ABA-responsive genes (*LTI65*, *LTP4*, *HVA22E*) and transcription factors (*ABI3*, *ABI4*, *ABI5*) that were repressed post-radicle protrusion, correlating with moisture increase, ROS accumulation and loss of desiccation tolerance (Smolikova et al., 2020). Furthermore, comparative RNA-Seq analysis of nodulated *vs* N-fed vetch *vs* pea under drought stress revealed that nodulated plants showed more attenuated response to drought with distinct regulation of specific TFs and metabolic pathways (Álvarez-Aragón et al., 2023). Similarly, other studies identified *DREB family* and SUMO ligases as major regulators under combined water stress (Henriet et al., 2019; Jarambasa et al., 2023). *WRKY* transcription factors such as *PsWRKY23, PsWRKY58* and *PsWRKY64* showing significant induction under abiotic stress (Kumar et al., 2024) and kinesin genes like *PsKIN8*, *PsKIN11* and *PsKIN54* associated with tissue specific and stress-responsive expression patterns in pea (Yuan et al., 2024). RNA-Seq revealed distinct molecular response to cold stress in tolerant and susceptible genotypes. Comparative studies between frost tolerant (Champagne) and sensitive (Terese) lines showed that chilling tolerance involved early activation of *CBF*, *COR* and *LEA* genes with faster induction in the tolerant genotype. While freezing tolerance in Champagne was associated with osmotic adjustment, antioxidant response, photosynthesis and hormone regulation (Legrand et al., 2013; Lucau-Danila et al., 2012). Further, transcriptomic analysis identified 4,981DEGs grouped into constitutive, chilling and freezing-responsive categories. Notably, many DEGs were genotype-specific and the tolerant genotype Champagne showed stronger constitutive and possibly more efficient cold tolerance mechanisms (Bahrman et al., 2019). Similarly, integrated sRNA and mRNA sequencing identified 39 miRNA–mRNA target pairs with inverse expression patterns and 11 conserved miRNAs in cold acclimation particularly frost-tolerant miR397-laccase module (Mazurier et al., 2022). In addition, genome wide identification of *PsZF-HD* gene family identified two major groups viz., MIF (*PsMIF1–PsMIF4*) and ZHD (*PsZHD1–PsZHD14*) involved in cold acclimation (Shi et al., 2023). Expression analysis revealed the upregulation of several *MADS-box* genes in response to drought and salinity (Gong et al., 2025). Pea plants exposed to different NaCl concentrations (0, 50, 75, and 100 mM) and water-deficit conditions at 100%, 75%, and 50% of field capacity to study the effects of water-deficit and salt stress. Genes related to flavonoids, proline and antioxidant enzymes (*PAL1, P5CR, POX and SOD*) were analyzed which showed *PAL1, P5CR* and *POX* genes increased expression under stress, while SOD expression decreased. Climax variety showed enhanced stress tolerance linked to higher *P5CR* and *PAL1* expression, while Meteor showed resilience through increased *POX* expression (Farooq et al., 2024).

Abiotic stress causes significant changes in plant proteome, viz., alteration in protein abundance, cellular localization, protein-protein interactions and modifications after transcription and translation (Kosová et al., 2018). Heat shock proteins (*HSPs*) and heat shock factors (*HSFs*) were first identified in 1962 (Kregel, 2002) are crucial for pea heat stress tolerance (Shah et al., 2020). Exposing pea plants to high temperatures (37°C for 6 hours) resulted in *HSPs* (22 kDa) low molecular weight accumulation (Wood et al., 1998). Various key *HSPs* viz., *chloroplast Pshsp26.2, mitochondrial Pshsp22.9* and *ER-localized Pshsp22.7* showed several thousand-fold increases in expression as compared to control seedlings at 42°C (Talalaiev and Korduym, 2014). Two ethylene response factors (*ERF95 and ERF97*) were identified that improve heat stress tolerance via the *EIN3-ERF95/ERF97-HSFA2* transcriptional cascade (Huang et al., 2023). Numerous other proteins play role in heat stress tolerance were reported (Ristic et al., 2008; Fu et al., 2012; Priya et al., 2024). A labelled free shotgun proteomic approach (nLC-MSMS) identified 367 proteins associated with primary metabolism and protein regulation across different genotypes under drought stress. Thus, suggesting that drought tolerance depends upon maintaining metabolic and protein protective functions, while susceptibility involves energy-intensive homeostatic processes (Castillejo et al., 2016). The various structural and protective proteins such as *seed biotin-containing protein* (*SBP65*), *tubulin alpha-1 chain*, and *vicilin* impart role in pea drought tolerance (Wang et al., 2012). In pea root nodules under moderate drought, different proteins were glycated through carboxymethylation which highlights role of glycation in regulating key processes like transport, protein degradation and metabolic pathways during drought stress (Julia et al., 2021). Salinity stress in pea also triggers proteomic changes, particularly in root tissues with key proteins like *pathogenesis-related (PR) 10*, SOD and *nucleoside diphosphate kinase (NDPK)* contribute to stress signaling and defense (Kav et al., 2004). Additionally, overexpression of *LecRLK* enhances salinity tolerance by improving Na+ compartmentalization, ROS scavenging, and cellular homeostasis, resulting in better growth without yield reduction (Vaid et al., 2015). Furthermore, the role of S-nitrosylation proteins in pea leaves under drought and salinity conditions revealed significant changes in S-nitrosylation patterns of peroxisomal proteins in response to stress (Ortega-Galisteo et al., 2012). Thus, these omics studies have advanced pea breeding through candidate gene identification, yet the functional validation lags due to pea’s complex, large, repetitive genome, uneven reference genome annotation assemblies and low transformation efficiency. Additionally, the stress tolerance traits are often highly environment dependent leading to inconsistent gene effects across varied environmental conditions. Multi-omics integration in pea is further constrained by weak transcript-protein correlations, limited temporal and spatial resolution of datasets, and the scarcity of comprehensive post-translational modification datasets. Addressing these constraints through pan-genome references, improving reference annotation, optimizing genotype-specific transformation and regeneration systems will strengthen the translation of genomic discoveries into the pea genetic improvement program for abiotic stress tolerance.

## 5 Next generation strategies for genomic improvement of abiotic stress tolerance

**Figure 2:**
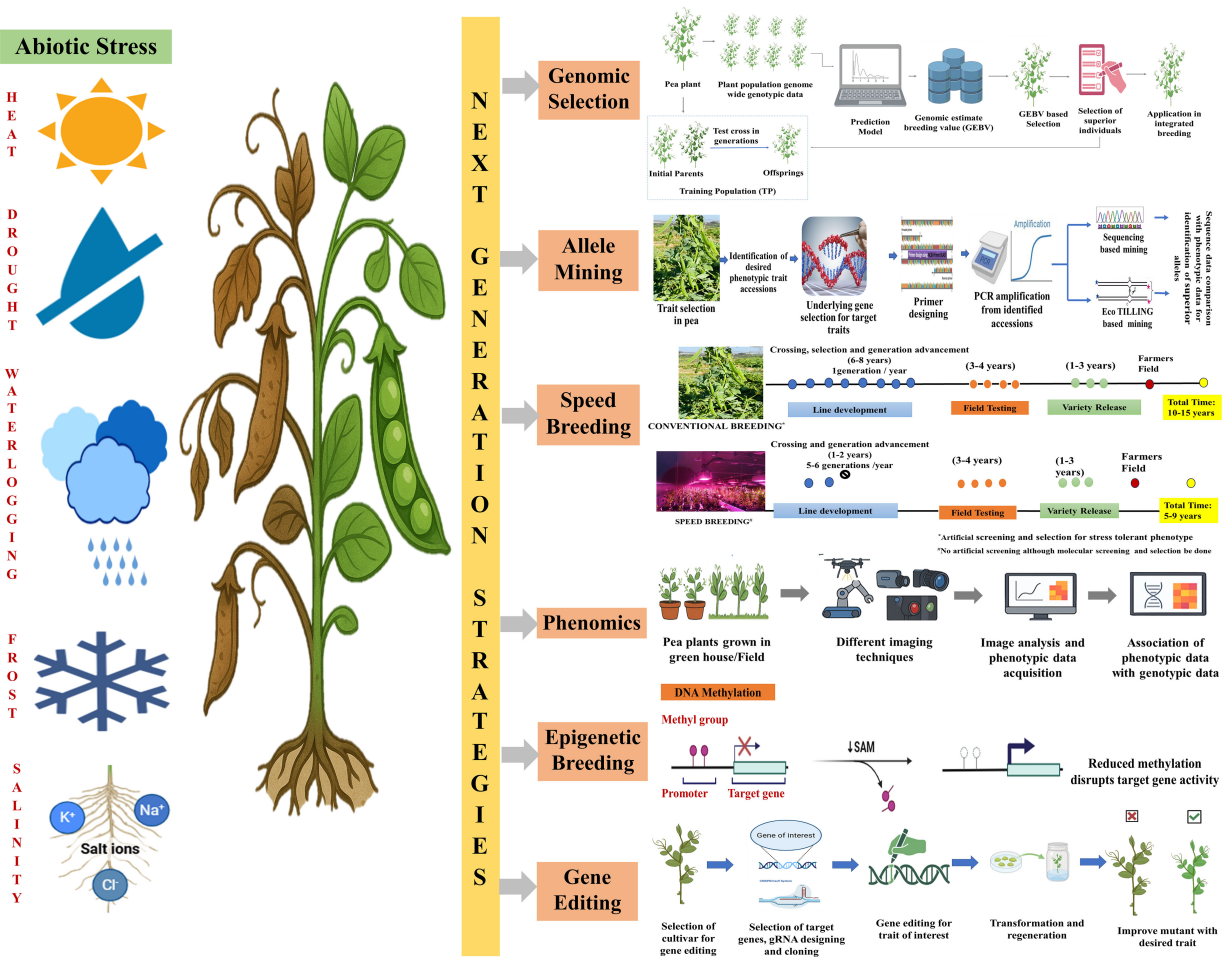
Next-generation strategies for enhancing abiotic stress tolerance in pea.

The integration of next-generation strategies along with the breeding and different molecular techniques has revolutionized the pea crop genetic improvement by allowing rapid and precise genetic enhancements in developing climate-resilient cultivars (**Figure 2**).

### 5.1 Genomic selection

Genomic selection (GS) uses genome-wide DNA markers to estimate breeding values (GEBVs) for complex polygenic traits (Meuwissen et al., 2001). This is frequently done using statistical models combining marker and phenotypic information from a different population. Briefly, two populations viz., training and testing are used (Jain et al., 2017). The key success of GS is genomic prediction which relies on accurate multilocation phenotyping data combined with high throughput genotyping data (Parihar et al., 2022). In pea, GS using six different methods (PLS, LASSO, SPLS, Bayes A, Bayes B and GBLUP) based on SNP array data showed good prediction accuracy for yield component traits and flowering time (Burstin et al., 2015; Tayeh et al., 2015c). GS models viz., Bayesian Lasso (BL) or ridge regression best linear unbiased prediction (rrBLUP) in pea under drought conditions outperformed the support vector regression (SVR) model, providing the best prediction with at least 400–500 markers (Annicchiarico et al., 2019). GS consistently outperformed phenotypic selection and evolutionary population methods and the lines derived from GS exhibited greater yield stability under stress prone conditions in pea (Tayeh et al., 2015c; Annicchiarico et al., 2025. Most of the GS applications for abiotic stress tolerance have been reported in cereal crops (Kumar et al., 2021; Lohithaswa et al., 2022) as compared to pea. Therefore, future research should integrate advanced genomic techniques like whole genome sequencing and machine-based prediction models with multi-omics data to unravel the biological pathways involved in different stress responses.

### 5.2 Allele mining

Allele mining identifies novel and superior alleles in wild germplasm which are then transferred from wild relatives into cultivated types (Gokidi et al., 2017). However, limited efforts have been made to mine superior alleles in peas. Various methods like sequencing, TILLING (Targeting Induced Local Lesions in Genomes) and EcoTILLING have been used to identify a specific gene’s sequence polymorphism (**Figure 2**; Gokidi et al., 2017; Selvakumar et al., 2023; Parihar et al., 2025). Allele mining has many applications, viz., functional marker development, gene diversity analysis, gene prediction, finding superior alleles and new haplotypes. However, there are various challenges like the collection of germplasm to be mined, inefficient TILLING primers and mutation load, which poses a major issue for allele mining utilization in pea breeding (Selvakumar et al., 2023). Wild germplasm is a rich source of promising under-utilized allele or allele combinations. For instance, a primary *Pisum* germplasm collection was studied to understand *Pisum* diversity and evolution (Jing et al., 2010) which clearly distinguishes the gene pools within the genus *Pisum*. Such studies offer a paradigm for characterizing *Pisum* germplasm worldwide and developing core collections (Upadhyaya et al., 2011). Allele mining venture in vegetable crop has also been started by Plant Genomics Research Unit (URGV) in France, offering screening for tomato (TOMATILL) and pea (PETILL) (http://urgv.evry.inra.frlUTILLdb). Furthermore, the increasing availability of NGS and recent pea genome assembly are expected to greatly expand the process of gene identification. Although the potential of these techniques for identification of stress tolerance alleles in pea breeding is limited so far (Selvakumar et al., 2023; Parihar et al., 2025). However, their strategic deployment can unlock the hidden genetic variation and usher into new era of precision breeding for abiotic stress tolerance.

### 5.3 Speed breeding

Peas usually take 100–120 days from sowing to maturity depending upon the cultivar. Traditional breeding methods aimed at developing climate-resilient cultivars are often time-consuming and labor-intensive and include long breeding cycles for segregating populations (F_2_, BC, RILs, NILs) development (Ahmad et al., 2021). To address these limitations, scientists are adopting a new innovative technique known as speed breeding (SB). This technique involves the manipulation of environmental conditions such as photoperiod and temperature around the crop to enhance photosynthesis, induce early flowering, accelerate seed maturation and ultimately reducing overall crop generation duration (**Figure 2**; Ghosh et al., 2018; Cazzola et al., 2020). SB facilitates rapid generation advancement (5–6 generations/year) which is especially valuable for pre-breeding, mapping population development and advancement of segregating generations (**Figure 2**). Standardization of speed breeding protocol in pea utilizes extended photoperiods (22-hour photoperiod) with supplement lighting (Light emitting diode) and precise temperature regulation (12-h 22°C/17°C). The pea generation time was significantly reduced to 6.9 (Ochatt et al., 2002) and (Mobini and Warkentin, 2016; Cazzola et al., 2020) generations per year (Ghosh et al., 2018). Plant development remained normal, successful crosses were achieved and seed germination rates were high under these rapid growth conditions (Ghosh et al., 2018). Recent advancements in pea demonstrated speed breeding efficacy in reducing the seed-to-seed cycle. So far, only two pea varieties have been developed using speed breeding protocol by University of Queensland, Australia and John Innes Centre, UK (Chaudhary and Sandhu, 2024). Speed breeding effectively accelerates generation advancements in pea. However, multi-environment testing of advanced generation remains essential for precise screening and phenotyping under field conditions. Therefore, integrating speed breeding with multi-environment field trials validates G×E interactions and ensures yield stability for stress-tolerant pea varieties.

### 5.4 Phenomics

Phenotyping has emerged as the primary bottleneck in plant breeding with the rapid advancement of genomics technologies (Pandey et al., 2021). Traditional phenotyping methods are often labor-intensive, time-consuming and prone to human error which limits breeders’ ability to evaluate large populations effectively. Phenomics platforms significantly accelerate breeding programs by providing large-scale accurate data on pea improvement. Various imaging techniques, viz., visible light imaging, infrared imaging, X-ray computed tomography, fluorescence imaging, and hyperspectral imaging produce multidimensional and distinctive phenotyping data when integrated with a strong software system (Sozzani et al., 2014). Using color imaging technology, an automated phenotyping tool was employed to analyze early vigor traits in *Pisum sativum var arvense* under controlled conditions [Plant Phenomics Victoria, Horsham (Nguyen and Kant, 2018). Further, *Pisum sativum var arvense* ability to withstand cold was also assessed using PlantScreen (Photon Systems Instruments, Brno, Czech Republic) and digital color imaging technology (Humplík et al., 2015). Additionally, the utilization of deep learning algorithms along with real-time aerial imaging, portable X-ray CT scanners and infrared imaging devices allows breeders to non-invasively evaluate the internal root architecture or nutrient distribution traits crucial for abiotic stress tolerance especially at early-stage field phenotyping in breeding programs.

### 5.5 Epigenetic breeding

Abiotic stress can cause alterations in the non-coding RNAs, DNA methylation and histone modifications activity. These modifications show fast and reversible gene expression changes, enabling plants to withstand stress without changing the primary DNA sequence (Sharma et al., 2022; Alfalahi et al., 2022). Furthermore, stress induced epialleles provide adaptive expression variation that can be selected and stabilized during the line development (Kumar et al., 2011). Additionally, these changes enable plants to develop “stress memory,” thereby enhancing their response to repeated stress.

These epigenetic variations can sometimes be heritable and can be used as selection targets in molecular assisted breeding (Ashapkin et al., 2020). Epigenetic breeding leverages stable epiallelic by selecting lines with stress-induced regulatory modifications through phenotyping in controlled stress environments. However, where genetic resistance remains scarce in pea, targeted epigenome editing using CRISPR dCas9 fused to methyltransferases create novel epi-alleles which can be introgressed into elite backgrounds via recurrent selection. Water deficit conditions induced cytosine methylation in pea root tip DNA compared to adequately watered plants (Labra et al., 2025). Further, epigenomic profiling using four key histone modifications (*H3K27me3, H3K4me3, H3K9me2* and *H3K9ac*) identified a novel stress adaptive bivalent chromatin state (*H3K9ac-H3K27me3*) that regulates metabolic and salinity responsive pathways (Wan et al., 2024) yielding stable epi-regions or epi-linked markers for selection during line advancement. These advances (Epigenetic assisted selection and editing) strengthen epigenetic pea abiotic stress tolerance breeding.

### 5.6 Genomic modification: genome and genetic engineering

Genetic engineering enables transgenic crop development with enhanced stress resistance by modifying specific genes. Over the past two decades, it has been used to improve abiotic stress tolerance in vegetable crops (Parmar et al., 2017). In pea, the first successful report on gene transfer utilizing *Agrobacterium* was reported by De Kathen and Jacobsen, where *nptlI* and *hpt* resistance genes were successfully integrated and expressed (De Kathen and Jacobsen, 1990). Since then, various researchers have made modifications to improve the transformation process. However, transformation efficiency remains below 5% and often ranges between 1-3% (Somers et al., 2003). Efforts have been made to overcome these hurdles by optimizing protocols for gene delivery and regeneration systems (Aftabi et al., 2017). A selection medium containing phosphinothricin and 4.5 μM zeatin improved regeneration efficiency, resulting in a transformation efficiency of 7.89% with a 90-minute infection time and 2 days of co-cultivation (Aftabi et al., 2017). Different abiotic stress gene transfer studies have been conducted in legumes. For instance, the *coda* gene enhances drought tolerance in chickpea (Sharmila et al., 2009). While Arabidopsis transcription factors (*AREB1* and *DREB2A*) expression (Sharmila et al., 2009; Barbosa et al., 2013) and RNAi suppression of *RACK1* gene (Li et al., 2018) improved drought tolerance in soybean. *A. thaliana* gene Na+/H+ overexpression in peas improved salt tolerance (Ali et al., 2015). Phenotypic evaluation of *AtNHX1* transgenic pea plants also revealed frost tolerance, though further investigation is required to understand the mechanism (Ali et al., 2018). Furthermore, *i*ncorporation of *Arabidopsis HsfA1d* by using expression vector pGWB415 increases fivefold expression of *HsfA1d* in pea-transformed plants under heat stress (42°C). However, this approach involves random gene insertion along with raising concerns over stability. Contrarily, GE technique enables researchers to modify the plant DNA by adding, removing or altering genetic material at specific sites within the genome (Komor et al., 2017). Various GE techniques are utilized with greater accuracy and efficiency than conventional breeding methods for abiotic stress tolerance in vegetable crops (Sardar, 2023). Genes within stress-associated gene regulatory networks, signal transduction and metabolic processes may be targeted via CRISPR technology to develop stress-tolerant crops (Kumar et al., 2023). In legumes, two genes viz., *RVE7* and *4CL* linked to drought tolerance in chickpea were knocked out using CRISPR/Cas9 mediated GE (Badhan et al., 2021). However, no studies have yet reported using CRISPR techniques for developing abiotic stress-tolerant mutants in pea. In pea, this technique has been used to edit genes viz. *phytoene desaturase* (*PDS*, Li et al., 2023), *Lipoxygenas* [*LOX*, Bhowmik et al., 2023), *β‐amyrin synthase* (*BAS*, Hodgins et al., 2024) and *Mitogen-activated protein kinases* (*MAPK6*, Kantsurova et al., 2024). These studies showcased the potential of CRISPR Cas9 to target genes involved in stress response pathways. Thus, the utilization of cutting-edge techniques for developing stress-tolerant pea mutants paves the way for a new era of plant breeding.

## 6 Conclusion and future prospects

Enhancing abiotic stress tolerance in pea is crucial for sustaining productivity under growing climate change challenges. Developing stress tolerant cultivars will stabilize yields and expand cultivation into non-traditional regions. The most promising strategies for immediate genetic gains include targeted integration of high-throughput phenomics for multi-environment stress screening, genomic selection models incorporating G×E effects to pyramid QTLs from landraces/wild relatives and adoption of speed breeding for rapid generation advancement. These strategies can enhance the efficiency and precision of pea breeding programs. However, lack of robust stress phenotyping methods and limited functional validation of candidate genes coupled with low transformation efficiency for genome editing in pea continues to constrain progress. Overcoming these requires validation of omics-driven gene discoveries, integration of precise phenomics with genomics-assisted pre-breeding and strengthening coordination between gene banks and breeding programs. A multidisciplinary framework involving geneticists, breeders, molecular biologists, and bioinformaticians will translate these advances into field level solutions, ensuring sustainable pea production amid climate challenges.
